# Modified β-Cyclodextrin Inclusion Complex to Improve the Physicochemical Properties of Albendazole. Complete *In Vitro* Evaluation and Characterization

**DOI:** 10.1371/journal.pone.0088234

**Published:** 2014-02-14

**Authors:** Agustina García, Darío Leonardi, Mario Oscar Salazar, María Celina Lamas

**Affiliations:** 1 IQUIR-CONICET, Facultad de Ciencias Bioquímicas y Farmacéuticas, UNR, Rosario, Santa Fe, Argentina; 2 Área Técnica Farmacéutica, Facultad de Ciencias Bioquímicas y Farmacéuticas, UNR, Rosario, Santa Fe, Argentina; 3 Área Farmacognosia, Facultad de Ciencias Bioquímicas y Farmacéuticas, UNR, Rosario, Santa Fe, Argentina; Hungarian Academy of Sciences, Hungary

## Abstract

The potential use of natural cyclodextrins and their synthetic derivatives have been studied extensively in pharmaceutical research and development to modify certain properties of hydrophobic drugs. The ability of these host molecules of including guest molecules within their cavities improves notably the physicochemical properties of poorly soluble drugs, such as albendazole, the first chosen drug to treat gastrointestinal helminthic infections. Thus, the aim of this work was to synthesize a beta cyclodextrin citrate derivative, to analyze its ability to form complexes with albendazole and to evaluate its solubility and dissolution rate. The synthesis progress of the cyclodextrin derivative was followed by electrospray mass spectrometry and the acid-base titration of the product. The derivative exhibited an important drug affinity. Nuclear magnetic resonance experiments demonstrated that the tail and the aromatic ring of the drug were inside the cavity of the cyclodextrin derivative. The inclusion complex was prepared by spray drying and full characterized. The drug dissolution rate displayed exceptional results, achieving 100% drug release after 20 minutes. The studies indicated that the inclusion complex with the cyclodextrin derivative improved remarkably the physicochemical properties of albendazole, being a suitable excipient to design oral dosage forms.

## Introduction

Cyclodextrins (CDs) are cyclic oligosaccharides consisting of six, seven or eight linked D-glucopyranose units (α, β and γ -CDs, respectively). Due to the chair conformation of the glucopyranose units, CDs molecules have torus macro-ring shape, like a truncated cone, with a hydrophobic cavity and a hydrophilic surface [1]. CDs are well known to form inclusion complexes with a variety of drugs resulting in the improvement of aqueous solubility [2]. Therefore, CDs have been extensively studied to improve certain properties of the drugs, such as aqueous solubility of hydrophobic drugs avoiding the use of organic solvents, surfactants, or lipids, to increase dissolution rates and oral bioavailability [3]. Additionally, CDs can also reduce gastrointestinal and ocular irritation, eliminate unpleasant odors or taste, and prevent drug-additive interactions [4], [5], [6], [7].

The enhancement of drug activity or the reduction of side effects can be achieved by inclusion complex formation through a molecular encapsulation process. The CD complexation enables the development of formulations for drugs that are difficult to formulate and deliver with the existing pharmaceutical excipients. To extend the functions of pharmaceutical additives, the combination of molecular encapsulation with other carrier materials is also an effective and a valuable tool in the improvement of drug properties.

Numerous chemical modifications of the CDs have been synthesized and novel derivatives are constantly being developed. Since each CD hydroxyl group differs in its chemical reactivity, the random substitution produces an important increase in solubility due to the decrease in the crystalline state and may also be helpful to overcome the formation of kidney damaging crystalline cholesterol complexes and also to achieve efficient drug delivery systems [8].

Albendazole (ABZ), methyl [5-(propyl-thio)-1-*H-*benzimidazole-2yl] carbamate, is a benzimidazole derivative with a broad anthelmintic spectrum [9]. ABZ is useful against several gastrointestinal parasites such as those producing hydatidosis. ABZ, belongs to the class II in the Biopharmaceutics Classification Systems (BCS). It is a poorly water soluble (1 µg/mL) and highly lipophilic drug (log *P* of 2.55), presenting low or erratic bioavailability leading to a variable oral absorption (≤5%) [10].

After oral administration, a drug must dissolve in the gastric fluids, in order to be absorbed into the systemic circulation. This process determines the rate and degree of absorption [11]. Several works have been carried out to improve the aqueous solubility and dissolution rate of ABZ. In this context it is worth mentioning, the preparation of solid dispersions [12], micellar drug delivery systems [13], microencapsulation [14], nanosuspensions [15], nanosize liposomes [16], ternary systems with polymers and CD [17] and inclusion complexes with CDs [18].

Particularly, when CDs were used to form inclusion complexes, different methods have been employed to characterize the interactions between guest molecules and CD, such as nuclear magnetic resonance (NMR) conductometric titration, potentiometric, spectrophotometric and fluorometric techniques, among others [19], [20], [21], [22].

However, in some instances, there are uncertainties regarding the complex stoichiometries and the lack of structural information using these techniques [23]. Electrospray ionization mass spectrometry (ESI-MS) is a powerful means of studying reaction products and non-covalent complexes between “host-guest” with high sensitivity and rapidity, at a very low level of sample consumption [24], [25], [26].

The characterization of the CD derivative and the inclusion complex is extremely important to understand the host-guest molecule interaction. Different techniques, such as ESI-MS and NMR spectroscopy (^1^H-^13^C Heteronuclear Single Quantum Correlation, HSQC, Heteronuclear Multiple Bond Correlation, HMBC and Rotating-Frame Overhauser Effect Spectroscopy, ROESY) were used to thoroughly investigate the synthesis reaction and to study the structural characteristics of supramolecular aggregates, which exhibited distinct properties from both “guest” and “host” molecules. The stoichiometry and equilibrium constants for complex formation, which allowed a quantitative description of the solubility behavior, were determined by phase-solubility studies.

Thus, the aim of this work was to improve a simple methodology to synthesize a β-CD citrate derivative (C-β-CD), to analyze their ability to form complexes with ABZ and to evaluate the solubility and dissolution rate of ABZ, the first chosen drug to treat gastrointestinal helminthic infections, one of the major health problems in many developing countries.

## Materials and Methods

### Materials

ABZ, β-CD, hydroxypropyl β-CD, methyl β-CD were supplied by Sigma-Aldrich Chemie GmbH (Steinheim, Germany).

All other chemicals were of analytical grade.

### Methods

#### Synthesis of β-CD citrate derivative

The C-β-CD was synthesized based on the reports written by S. Chaleawlert-umpon et al. with minor modifications [27]. Citric acid (CA) (10.57 mmol, 2.03 g) was dissolved in water (1.2 mL), and followed by the addition of β-CD (1.76 mmol, 2.00 g). The molar ratio of β-CD and CA was 1∶6. The reaction mixture was refluxed at 100°C for 6 h and samples were taken off at 1, 2, 3, 4, 5 and 6 h to follow the progress of the reaction. Then the product (C-β-CD) was precipitated employing isopropanol and centrifuged at 6000 rpm. The precipitate was washed with isopropanol to remove un-reacted CA. The procedure was repeated until the pH of supernatant was neutral. The nonappearance of free CA was also confirmed by ESI-MS. Finally, the product was dried 24 h at 60°C.

The reaction progress was followed by two methods: ESI-MS analysis and measuring the total carboxyl groups by titration [27], [28].

- **ESI-MS analysis.** High Resolution Mass spectra were recorded on a Bruker micrOTOF-Q II spectrometer (Bruker-Daltonics, Bremen, Germany). MS and MS-MS were carried out as followed: source type: ESI, ion polarity: positive and negative, scan begin: 200 m/z, scan end: 2000 m/z, set nebulizer: 0.4 bar, set dry heater: 180 °C, set dry gas: 4.0 L/min, set capillary: 4500 V, set end plate offset: -500 V, set collision Cell RF: 1000–1500 Vpp. Collision energy for MS experiments: 35 eV.

Specifically for low molecular weight acidic molecules such as CA, it has been used the negative ion, tune low mode, under the following conditions, set collision Cell RF: 150.0 Vpp [29].

All the samples were dissolved in formic acid (0.4% v/v) and then diluted with water and methanol.

- **Total carboxyl content of C-β-CD.** The total amount of carboxyl groups exhibited in the final product was determined by acid-base titration, based on previous 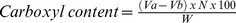 works [22], [30]. An accurate amount of C-β-CD was added to a conical flask containing an excess of 0.1 M NaOH solution and stirred for 1 h. The C-β-CD was completely dissolved, then, the NaOH was titrated with 0.1 N HCl solution employing phenolphthalein as an indicator [30]. The total amount of carboxyl groups of C-β-CD was calculated as follows:
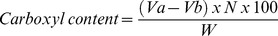

where N is the normality of HCl (eq/L), Vb is the volume of HCl without sample (mL), Va is the volume of HCl in presence of sample (mL), and W is the weight of sample (g).

#### Solubility diagrams

Solubility diagrams were obtained according to Higuchi and Connors [31]. Briefly, excess amounts of ABZ (30 mg) was added to 10 mL of distilled water (pH 6.3) containing various concentrations (0.60 mM) of β-CD, hydroxypropyl β-CD (HP-β-CD), methyl β-CD (M-β-CD) and the synthesized C-β-CD. Samples were shaken for 72 h in an elliptical shaker at 180 rpm and 37°C and then filtered through a 0.45 µm membrane filter (Advantec MFS, Inc.). ABZ concentration in the filtrate was spectrophotometrically analyzed at 291 nm (Boeco S-26 spectrometer, Hamburg, Germany). The phase-solubility diagram was therefore obtained and the complex formation constant (*K*f) was calculated [32], [33] according to the equation: S (slope), S_0_ (solubility of the drug in the absence of CD)
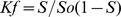



Each experiment was performed in triplicate.

#### Preparation of the inclusion complexes and physical mixtures

The inclusion complexes were prepared by spray drying (SD) method dissolving ABZ (0.56 mol) in acetic acid (10 mL). Then, C-β-CD (0.56 mol) and water (20 mL) were added to the solution. The resulting solution was spray-dried in a Mini Spray Dryer Buchi B-290 (Flawil, Switzerland) under the following conditions: inlet temperature: 130°C, outlet temperature: 70°C, air flow: 38 m^3^/h, feed: 5 mL/min, and aspirator set: 100%.

Additionally, physical mixtures (PM) between C-β-CD and ABZ were prepared in a mortar by mixing the drug and carrier for 10 minutes.

### C-β-CD and Complex Characterization

#### Fourier transform infrared spectroscopy

Fourier transform infrared (FT-IR) spectra were obtained by an FT-IR-Prestige-21. Shimadzu (Tokyo, Japan) using the KBr disk method (2 mg sample in 100 mg KBr). Scanning range was 450 to 3,900 cm^−1^ with a resolution of 1 cm^−1^.

#### Differential Scanning Calorimetry

Differential scanning calorimetry (DSC) was performed on a Shimadzu TA-60 (Kyoto, Japan) calorimeter, using 5 mg samples in crimped aluminium pans. The instrument was calibrated using indium and zinc as standards. Nitrogen was used as a purge gas and an empty aluminium pan was used as a reference. Each sample was scanned at a rate of 5°C/min from 25 to 350°C, under N_2_ atmosphere (flow rate 30 mL/min).

#### X-Ray Diffraction

Data collection was carried out in transmission mode on an automated X’Pert Phillips MPD difractometer (Eindhoven, The Netherlands).

X-ray diffraction (XRD) patterns were recorded using CuKα radiation (λ = 1.540562 Å), a voltage of 40 kV, 20 mA current and steps of 0.02° on the interval 2*θ* = 10°–40°. Low peak broadening and background were assured by using parallel beam geometry by means of an X-ray lens and a graphite monocromator placed before the detector window.

Data acquisition and evaluation were performed with the Stoe Visual-Xpow package, Version 2.75 (Germany).

#### ESI-MS Analysis

The stoichiometry of the complex was confirmed by mass spectrometry with high resolution; the equipment used was a micrOTOF-Q II spectrometer, Bruker-Daltonics (Bremen, Germany). To perform this analysis 1 mg of ABZ and an excess of C-β-CD were dissolved in 1 mL of formic acid (0.4% v/v), and then diluted 1/100 with methanol: water (50∶50). These studies were performed under the same conditions followed in *ESI-MS analysis*. For the MS-MS analysis, 30 eV energy of fragmentation was used.

#### NMR Spectroscopy

A complete NMR study (HSQC, HMBC and CIGAR-HMBC spectra) was conducted to determine the β-CD position modification.

#### ROESY Experiments

Two-dimensional rotating-frame Overhauser effect epectroscopy (ROESY) experiments were used to confirm the current complexation of ABZ with the C-β-CD, as well as to characterize their binding mode. ABZ:C-β-CD complex (10 mg) was solubilized in 0.5 mL 0.1 N DCl in D_2_O, and filtrated (0.45 µm Millipore membrane filter).

ROESY measurements were performed with a Bruker Avance 300 instrument (Karlsruhe, Germany) with a 5 mm probe using the roesyph pulse sequence (Bruker) with the experimental conditions as follows: 32 scans, acquisition time 0.222 s, pulse delay 1.92 s and 512 data points [34]. The resonance at 4.7 ppm was used as internal reference due to residual solvent water (H_2_O and HDO).

#### Dissolution Studies

Dissolution studies were performed in 900 mL HCl 0.1 N at 37°C, according to U.S. Pharmacopeia (USP) Apparatus 2 (SR8 8-Flask Bath, Hanson Research, Chatsworth, CA) with paddle rotating at 50 rpm [35]. Samples of ABZ pure drug, PMs and SDs equivalent to 100 mg of the drug were spread on the surface of the dissolution medium and the time 0 was recorded. At appropriate time intervals, 5 mL of samples were withdrawn, and filtered (pore size 0.45 mm). The amount of drug released was determined by UV analysis by measuring the absorbance spectrophotometrically at 291 nm.

## Results and Discussion

### Reaction Progress

**Figure 1:**
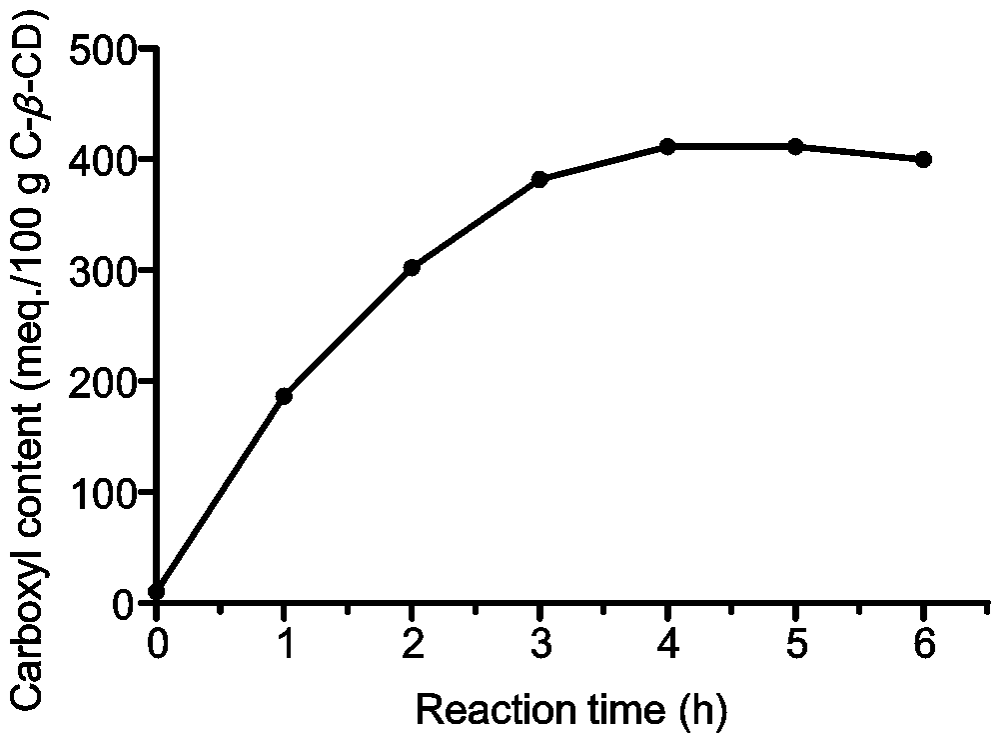
Reaction progress. Total amount of carboxyl groups vs time.

To evaluate the progress of the synthesis of the CD derivative, the total carboxyl content of C-β-CD at different reaction times was analyzed by acid-base titration (Figure 1). A maximum of carboxyl groups content was reached after a 4 h reaction.

Additionally, to corroborate the β-CD derivative reaction progress, samples were assayed by means of ESI-MS analysis. The analysis in negative ionization mode allowed observing a molecular ion with m/z 1307.3. The signal of β-CD derivative appears at 1307.3 and the β-CD starting material at m/z 1133.3. These results confirmed the degree of substitution of C-β-CD by the calculation of the molecular weight.

Meanwhile 4 h of reaction progress, the intensity of the signal of the β-CD starting material continuously decreased while the signal of the product was increased. After 4 h of the running chemical reaction no changes in the spectrum were observed. This result was consistent with that obtained by carboxylic titration.

Furthermore, it was also verified by ESI-MS analysis the nonappearance of un-reacted CA. These spectra were done under negative ion, tune low mode conditions to stabilize low molecular weight ions.

### Solubility Diagrams ABZ:CDs

**Figure 2:**
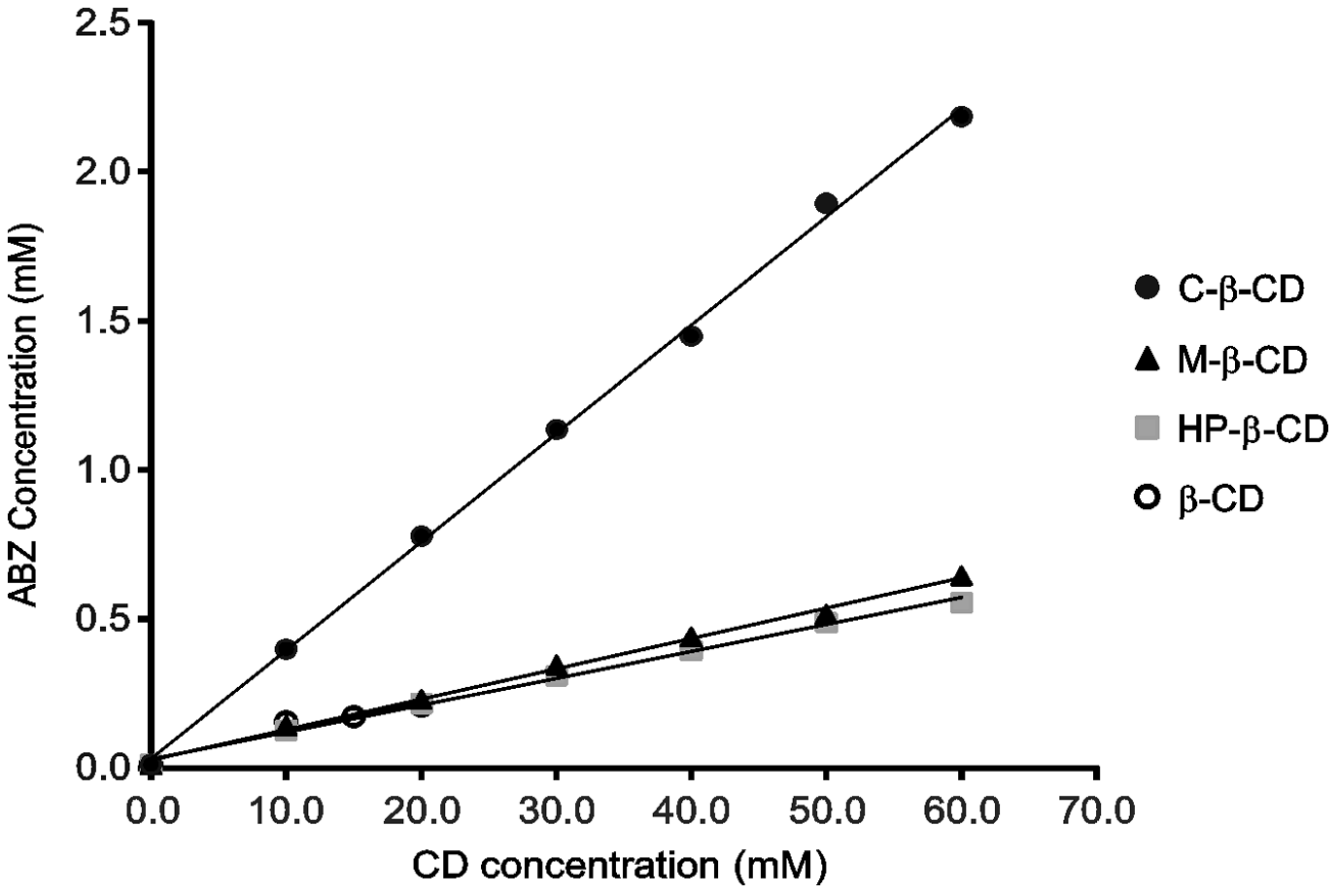
Phase solubility diagram. Concentration of ABZ against increasing concentrations of CDs.

The phase solubility diagrams of ABZ:β-CD, ABZ:HP-β-CD, ABZ:M-β-CD, ABZ:C-β-CD are represented in Figure 2.

The corresponding complex formation constants (*K*f) showed the relationship between ABZ and the CDs, fitting an A_L_-type system very well [31]. This type of curves indicated the formation of soluble 1∶1 type inclusion complex. The *K*f value obtained for the inclusion complex between ABZ and C-β-CD was 1037.34 M^−1^, suggesting a relatively stable complex in comparison with the *K*f obtained with traditional CDs (β-CD: 67.90 M^−1^; HP-β-CD: 313.16 M^−1^; and M-β-CD: 336.70 M^−1^). It could then be suggested that the stability of the complex ABZ:C-β-CD could be due to an interaction between the acidic groups of the derivative and the basic groups of ABZ.

### C-β-CD and Complex Characterization

#### FT-IR

**Figure 3:**
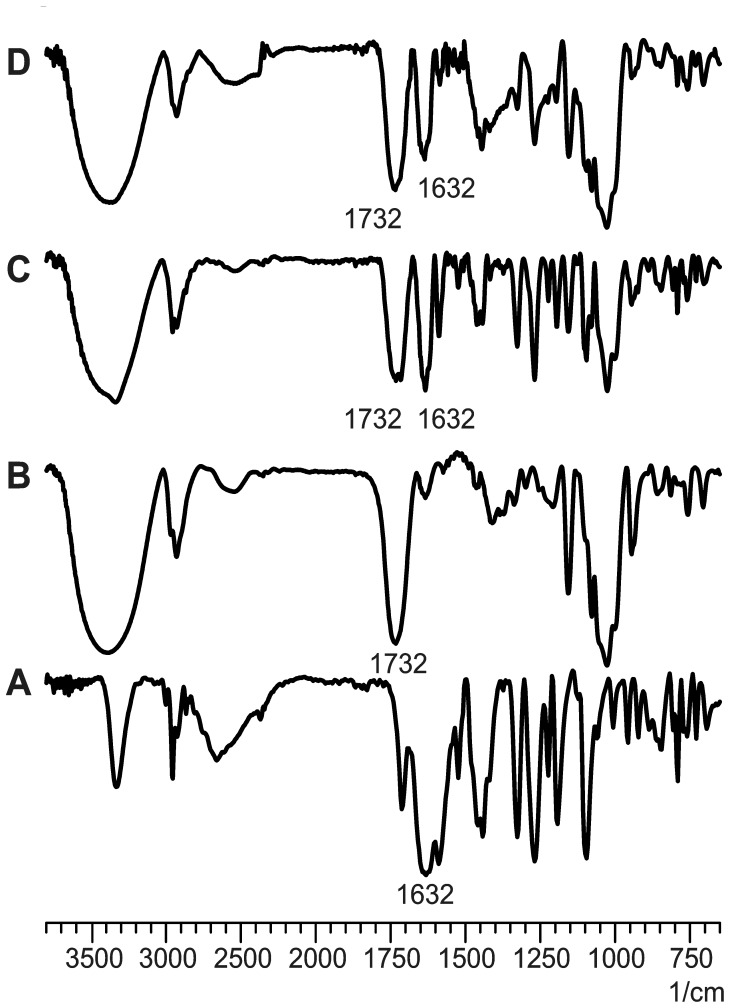
FT-IR spectra. A: ABZ (pure drug), B: C-β-CD, C: ABZ:C-β-CD obtained by PM, D: ABZ:C-β-CD obtained by SD.

The FT-IR spectra of ABZ, C-β-CD and systems ABZ:C-β-CD obtained by PM and SD techniques are represented in Figure 3. The C-β-CD showed a peak at 1732 cm^−1^ corresponding to an ester group (which was formed by the reaction between carboxyl groups of the CA and hydroxyls groups of the β-CD). The spectrum of ABZ:C-β-CD (PM) clearly exhibited the peaks corresponding to the samples of ABZ and C-β-CD and no shifting could were observed. On the other hand some peaks could be detected in spectrum D in accordance with ABZ patterns (1632 cm^−1^ corresponding to the carbonyl group). This fact could indicate the decrease in the crystallinity of the solid structure of ABZ loaded in the inclusion complex prepared by SD.

#### Differential Scanning Calorimetry

**Figure 4:**
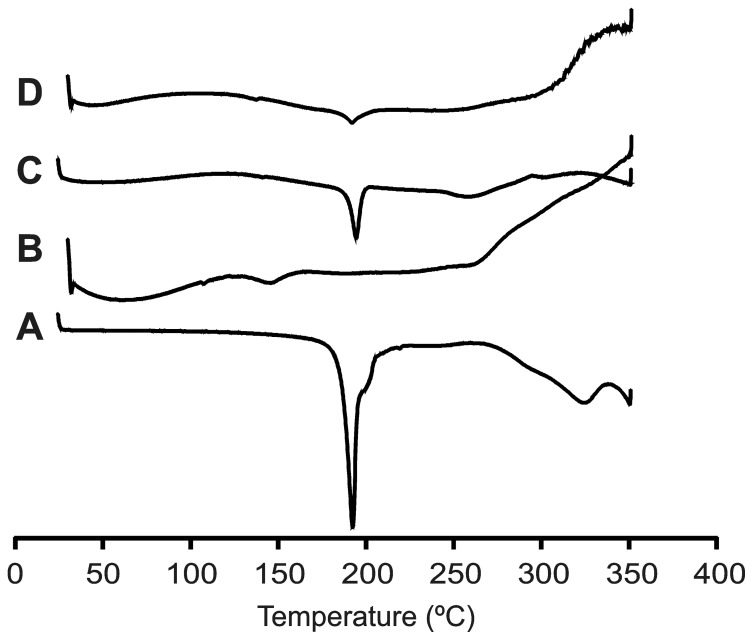
DSC thermograms. A: ABZ (pure drug), B: C-β-CD, C: ABZ:C-β-CD obtained by PM, D: ABZ:C-β-CD obtained by SD.

DSC thermograms of ABZ, C-β-CD and ABZ:C-β-CD prepared by PM and SD are shown in Figure 4. The characteristic sharp melting peak of ABZ is observed at 196.84 °C. The intensity of the drug endothermic peak was also exhibited in the system obtained by PM. In this particular case, the smaller intensity of the ABZ peak could be due to the dilution effect produced by the carrier ratio. The peak was also present in the system obtained by SD but showed less intensity. This decrease in the ABZ peak could be due to the structure of the C-β-CD and the SD process [36].

#### X-Ray Diffraction

**Figure 5:**
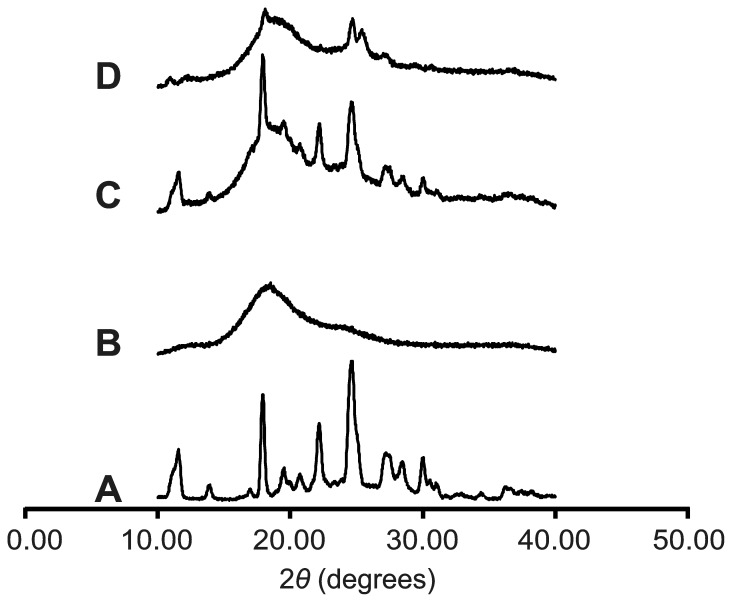
X-ray diffraction patterns. A: ABZ (pure drug), B: C-β-CD, C: ABZ:C-β-CD obtained by PM, D: ABZ:C-β-CD obtained by SD.

Figure 5 shows the XRD spectra of ABZ, C-β-CD and the systems ABZ:C-β-CD obtained by PM and SD. The derivative C-β-CD showed an amorphous structure; ABZ presented intense and characteristic peaks at 2*θ* 11.51; 17.85; 22.09 y 24.54. The diffraction patterns of ABZ in the PM were similar to the pure drug, indicating that the crystallinity of ABZ did not essentially change. The smaller intensity of some peaks of ABZ could be due to the dilution effect in the presence of the C-β-CD. On the other hand, diffraction patterns of SD systems showed fewer, broader, and less intense peaks, showing only two weak peaks of the drug at 2*θ* 17.85 and 24.54 which suggests that ABZ is in an amorphous state. Therefore, XRD analysis confirmed DSC results for the synthesized derivative inclusion complex could be attributed to the partial complexation of the drug in the CD cavity [37].

#### ESI-MS Analysis

**Figure 6:**
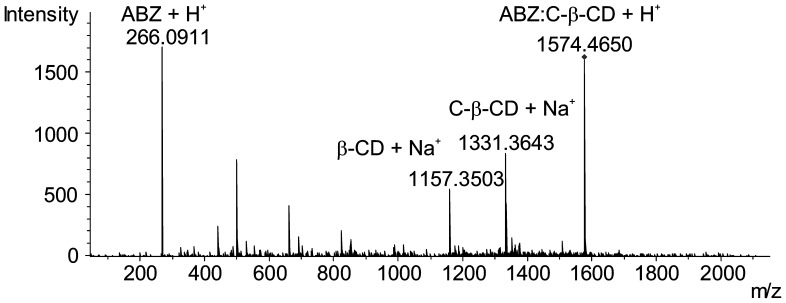
MS-MS spectrum. Product ion mass spectrum of the protonated molecules ABZ:C-β-CD.

Relative abundances and stoichiometries of the inclusion complex ABZ:C-β-CD formed by SD was determined using ESI-MS. The analysis in positive ionization mode allowed us to observe a molecular ion with *m/z* 1574.4650. This revealed that the inclusion complex was formatted in a ratio 1∶1 drug:carrier; which is in agreement with the results obtained from the solubility diagrams. No signals were observed with a ratio *m/z* related to other complex stoichiometries. Thereafter, the molecular ion was analyzed by MS-MS. The fragmentation of 1∶1 ABZ:C-β-CD is shown in Figure 6. The major molecular ions observed were: ABZ:C-β-CD (1574.4650); C-β-CD (1331.3643); β-CD (1157.3503) and ABZ (266.0911).

#### NMR Spectroscopy

After a complete NMR study conducted to determine the β-CD position modification, it was confirmed that the hydroxyl groups at the C6 and C2 position have been substituted by the CA.

Two dimensional NMR spectroscopy provides information through the observation of intermolecular dipolar cross-correlations. Closely located protons can produce a nuclear Overhauser effect (NOE) cross-correlation in NOE spectroscopy (NOESY) or ROESY. Spatial contacts within 0.4 nm from two species are indicated by the presence of NOE cross-peaks [38].

**Figure 7:**
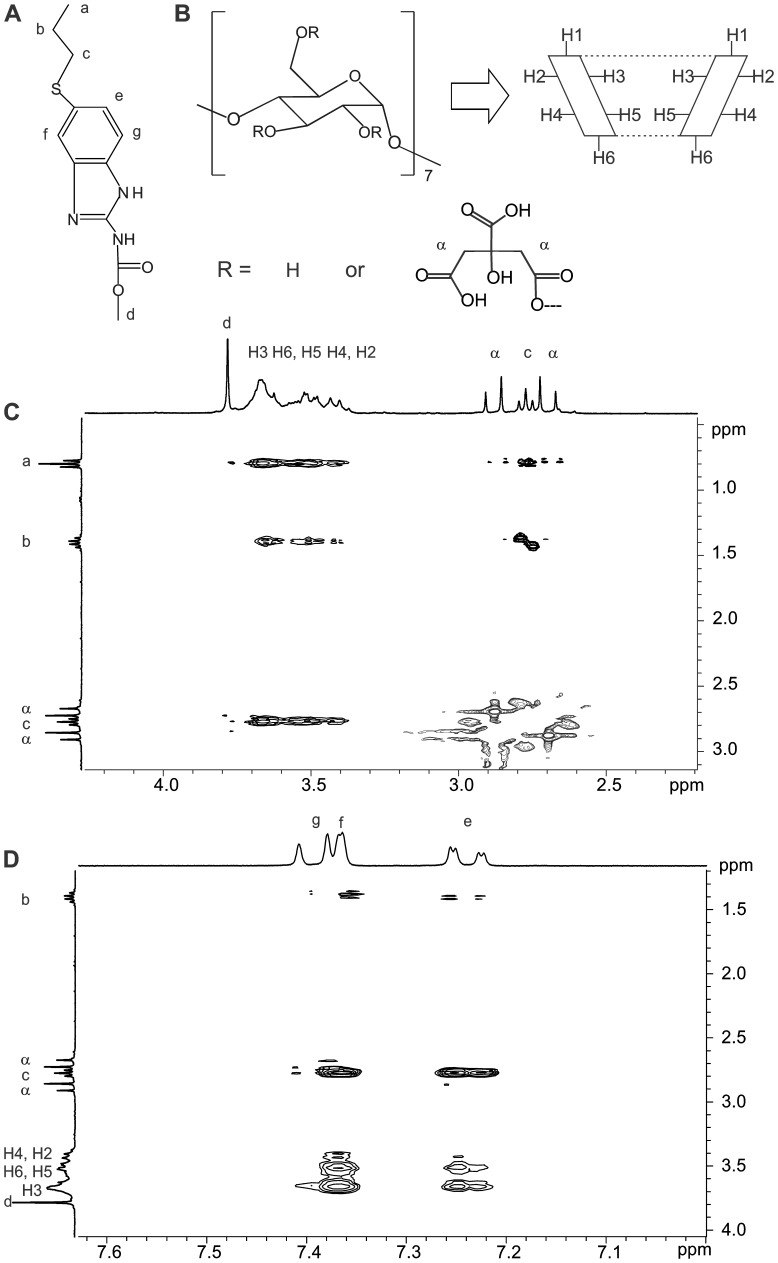
ROESY spectrum. A: ABZ proton labelling, B: C-β-CD proton labelling. C and D: Plot of two dimensional ROESY spectrum of ABZ in the presence of C-β-CD.

The protons labels of ABZ and C-β-CD and the ROESY spectrum obtained from the system ABZ:C-β-CD is shown in Figure 7. It was feasible to determine that the internal protons (H3, H5, H6) of C-β-CD were closer to protons of the tail of ABZ (a: δ = 0.80 ppm, b: δ = 1.40 ppm and c: δ = 2.79 ppm, Figure 7 C).

Additionally, the internal protons of the derivative C-β-CD presented cross-peak with the aromatic protons of ABZ (e: δ = 7.24 ppm, f: δ = 7.37 ppm and g: δ = 7.74 ppm). Finally the cross-peak between aromatic and tail protons of ABZ was observed in Figure 7 D.

Analyzing the results of ROESY spectrum, it could be concluded that both the aromatic ring and the tail of ABZ are inside the CD derivative and that the tail is probably folded over the aromatic ring.

#### Dissolution Studies

**Figure 8:**
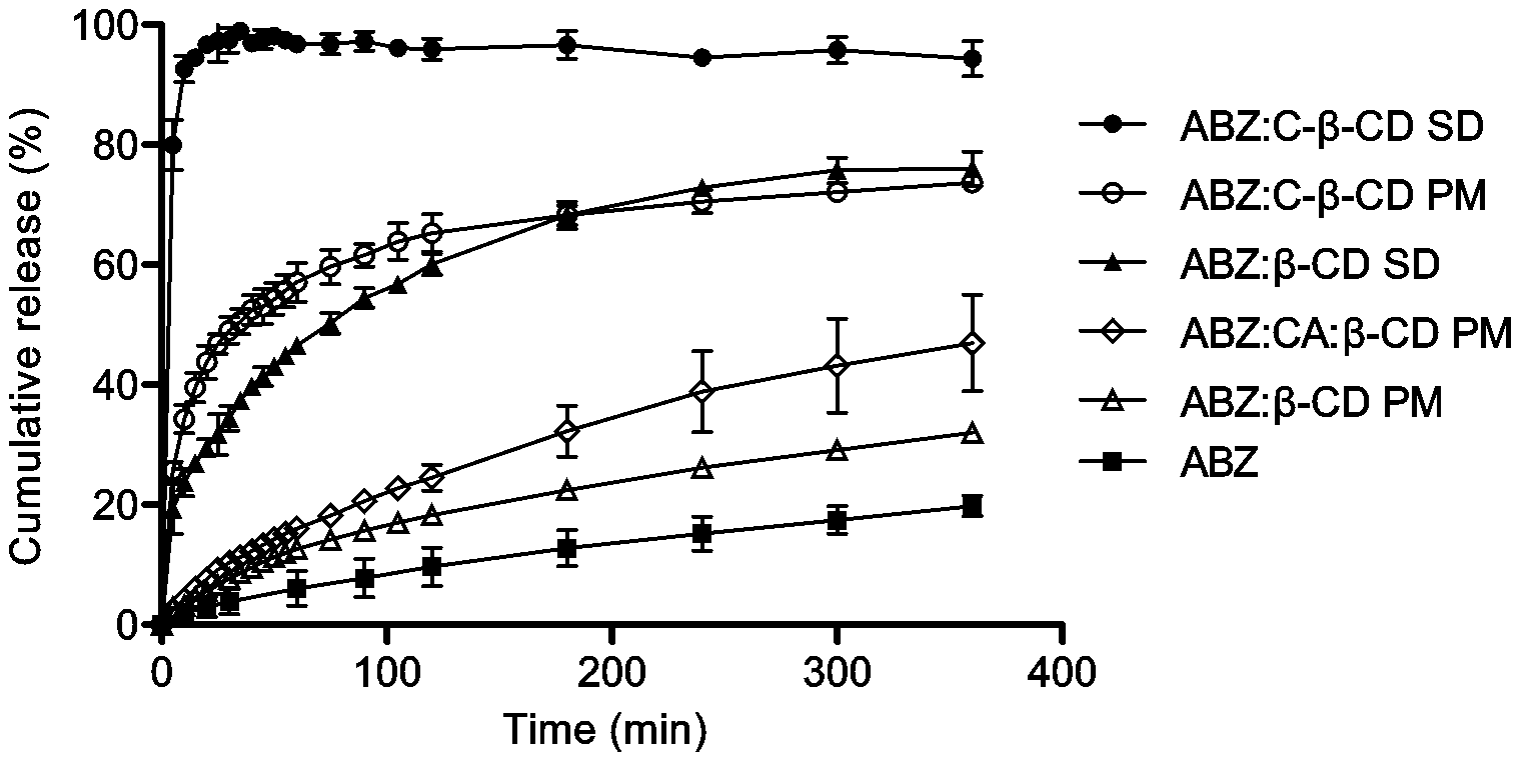
Dissolution profiles. Release profiles of ABZ (raw material), ABZ loaded in PMs, ABZ in the inclusion complexes (β-CD and C-β-CD). ABZ loaded in PMs (β-CD and CA). Test conditions: 0.1 N HCl, 37°C (n = 3, ±SD).

Dissolution profiles were performed to determine the dissolution rate of ABZ loaded in the systems ABZ:C-β-CD, ABZ:β-CD (obtained by SD or PM) and ABZ without any treatment (Figure 8). The profiles corresponding to ABZ:β-CD and the pure drug showed similar results, reaching nearly a 20% of drug release after six hours of the running assay.

The ABZ:β-CD (SD) complex and the PM prepared with C-β-CD and the drug achieved almost 80% of drug release after 6 hours.

The results obtained by the dissolution profiles of ABZ loaded in the inclusion complex with the C-β-CD prepared by spray drying exhibited the 100% drug release after 20 minutes of the running assay. These results remarkably demonstrate the improvement of the dissolution rate of ABZ, loaded in this synthesized derivative C- β-CD.

Furthermore, to evaluate the influence of CA in the drug release rate, it has been performed a PM (CA, ABZ and β-CD) dissolution profile. The CA amount was calculated based on the meq. of carboxyl groups in the C-β-CD. Figure 8 showed clearly that the CA did not affect the ABZ dissolution rate enhancement confirming the high potency of the C-β-CD as an excipient to improve remarkably the ABZ solubility and dissolution rate.

## Conclusions

In the present study, a β-CD derivative was synthesized. The reaction was followed efficiently by ESI-MS and the acid-base titration of the product. The derivative exhibited a high affinity for ABZ with a *Kf* 15.3 times higher than the traditional β-CD. The solubility diagrams presented type A_L_ curves indicating a 1∶1 ratio ABZ:C-β-CD complex. The ABZ loaded in the systems obtained by SD reflected a significant decrease in its crystallinity, which suggests a partial inclusion in the carrier. ESI-MS/MS-MS spectrum confirmed the stoichiometry of the complex and the ROESY assays showed that the tail and the aromatic ring of ABZ were inside the cavity of C-β-CD. The drug dissolution rate displayed exceptional results, achieving 100% drug release after 20 minutes.
